# Big Five Personality Traits and Compulsive Buying: The Mediating Role of Self-Esteem

**DOI:** 10.3390/ejihpe14010007

**Published:** 2023-12-29

**Authors:** José Manuel Otero-López, María José Santiago, María Cristina Castro

**Affiliations:** Department of Clinical Psychology and Psychobiology, Faculty of Psychology, C/Xosé María Suárez Nuñez, s/n, Campus Vida, 15782 Santiago de Compostela, Spain; mariajose.santiago@usc.es (M.J.S.); mariacristina.castro@usc.es (M.C.C.)

**Keywords:** personality, Five Factor Model, self-esteem, compulsive buying, path analysis

## Abstract

The inter-relationships between the Big Five personality traits, self-esteem, and compulsive buying are supported by strong empirical evidence. What is yet unknown is to what extent self-esteem can channel the influence of personality traits on compulsive buying. The main objective of this study is to explore the possible mediating role of self-esteem in the link between the Big Five personality traits and compulsive buying. Path analysis results, using a sample of 487 university students, generally confirm the suitability of the proposed model in which self-esteem mediated the effects of the Big Five personality traits (neuroticism, extraversion, agreeableness, openness to experience, and conscientiousness) on compulsive buying. Moreover, a direct effect of neuroticism and conscientiousness on compulsive buying was found. Finally, based on the finding that self-esteem acts as a necessary filter in the analysis of the five factors–compulsive buying relationship, several action-oriented guidelines for the prevention or intervention of this behavioral problem are suggested.

## 1. Introduction

Compulsive buying (CB), understood as a consumer’s tendency to be preoccupied with buying which is revealed through repetitive buying and a lack of impulse control over buying [1], has become an emerging public health issue in the last few decades. The cumulative evidence on its growing incidence and prevalence, particularly among the young [2,3,4,5], and its negative personal and social repercussions [6] stress the need for further research that contributes to the implementation of prevention and intervention measures with assurances of efficiency.

From a historical perspective, out of a wide range of variables that have been found to be linked to this phenomenon (of a personal, family, group, and contextual nature), it has been personality variables that have mostly drawn the attention of researchers and consequently the ones that have yielded the most findings [7,8]. Among other explanatory determinants, impulsivity [9], neuroticism [10], narcissism [11], the negative emotions of anxiety and depression [12,13], self-esteem [14], self-efficacy [15], social support [16], optimism [17], coping strategies [18] and generativity [19] have shown their predictive capacity for CB. 

Beyond the isolated study of personal determinants, an unprecedented interest in the analysis of CB has emerged in the last few years [20,21,22,23,24] within the context of a proposal with a high heuristic value in the field of personality psychology: the five factor model (FFM) [25]. This model is currently the most consensual and validated taxonomy of personality traits [26]. According to the FFM, “traits are organized hierarchically from narrow and specific to broad and general dispositions: neuroticism, extraversion, openness to experience, agreeableness, and conscientiousness constitute the highest level of the hierarchy” [26] (p. 165). Generally, these personality domains have been found to be useful in explaining many behavioral problems and/or addictions, including problematic Internet use [27], problematic smartphone use [28], problem gambling [29], Facebook addiction [30], exercise addiction [31], pornography addiction [32], Instagram addiction [33] and food addiction [34]. 

As far as the field of CB is concerned, although empirical evidence is limited, previous studies have found that the FFM traits have a critical role in explaining certain people’s predisposition to developing CB [7]. More specifically, the findings obtained from this model thoroughly confirm that whereas neuroticism is a major risk factor for CB [10,21,23,24,35,36,37,38,39,40,41], conscientiousness is a protection factor for this problem [21,22,23,37,39]. As for extraversion, most studies report positive, albeit modest, correlations with CB [23,41]. The findings for the remaining personality dimensions are far from concluding. Thus, in the case of agreeableness, some studies have reported a negative relationship with CB [35,39,40,42], whereas others have found a positive one [38,43,44]. As for openness to experience, once again, contradictory results have been found: positive influence has emerged in some studies [23,44,45] whereas some others have reported a negative influence in CB [36,43].

Beyond the documented impact of the personality variables in general and, specifically of the Big Five (BF) personality traits, on CB, one of the most important gaps in contemporary research is the identification of the mediating variables. The exhaustive review of empirical studies conducted in the field of CB shows that only materialism [38,46], hedonistic shopping experiences [23] and negative perfectionism [22] channel the influence of the BF on this behavioral problem. Another variable that, from the adjoining fields of CB, has recently attracted the interest of researchers with regard to its potential mediating role has been self-esteem. Indeed, it has been found that this variable channels the influence of the BF on smartphone addiction [47] and Internet addiction [48]. In the field of CB, however, no study has been conducted that looks into the role of self-esteem in the BF personality traits–CB link. And it is this very circumstance that has prompted this study, the purpose of which is to put forward a model of causal influences in which the BF personality traits are the exogenous variables, self-esteem is the mediating variable and CB is the endogenous variable. 

In our pursuit to provide a theoretical foundation to the causal model that we intend to verify (BF personality traits–self-esteem–CB) it should be noted that this model has its reference and is founded on the importance given to the self-concept (with self-esteem as its evaluative component) in *The Five-Factor Theory of Personality*—FFT [26]; specifically, from this theoretical framework, it is suggested that the BF basic tendencies of personality (represented by neuroticism, extraversion, agreeableness, openness to experience and conscientiousness) influence people’s self-conceptions, so that the perception or assessment that a person has about themselves can be shaped by traits.

Self-esteem, represented as an assessment aspect of self-concept and the five-factor theory of personality have been analyzed in different studies. The large study conducted by Robins et al. [49] is particularly relevant in this regard. Its authors point out that the set of the BF personality factors accounted for 34% of the variance in self-esteem and that the traits that were most strongly related to self-esteem were emotional stability, extraversion, and conscientiousness. More recently, Pilarska [50] concluded that all personality traits, except agreeableness, positively predicted self-esteem, with Emotional stability being the primary predictor. Furthermore, the review confirms consistent associations of self-esteem with each of the BF personality traits. Generally, a strong negative link between neuroticism and self-esteem is reported [51,52,53,54,55,56]. For other personality dimensions, positive associations with extraversion [52,57], conscientiousness [55,58], agreeableness [55,57] and openness to experience [57,59] have been reported. The two latter personality factors showed the weakest association.

As for the link between self-esteem and CB, many studies confirm that covariation is negative and significant [3,36,60,61]. Similarly, it has been documented that compulsive buyers as compared to buyers who do not present with this problem, score significantly lower in self-esteem [17,62,63,64]. Furthermore, low self-esteem has proved to be a significant predictor of CB [14,15,36,65]. 

In sum, despite the fact that the reviewed previous literature reports the existence of important links between the BF personality traits and CB, to our knowledge no study has looked into the mediating role of self-esteem in relation to the BF personality traits–CB. Consequently, the objective of this study is to analyze to what extent self-esteem channels the effect of the BF personality traits on CB. 

## 2. Materials and Methods

### 2.1. Procedure and Participants

This study is part of a wide spectrum research project, the objective of which is to gain insight into the role that different psychological variables play in CB in Galicia, Spain. Specifically, following a cross-sectional design, a convenience sample of students from the University of Santiago de Compostela was used. The application of different self-reports was performed by researchers, who were previously trained for field work, during class. Inclusion criteria for this research were as follows: being a fluent Spanish speaker, not currently (or in the last 6 months) undergoing psychopharmacological treatment or psychotherapy and having no other current impulse control disorder other than CB. The anonymity and confidentiality of the data were guaranteed. No incentives were offered for participation in this study.

The sample included in this study comprised 487 university students. A total of 214 were male (43.9%) and 273 were female (56.1%), with a mean age of 18.92 years (SD = 1.02). According to their field of study, the distribution of the sample was as follows: 29.1% Sciences, 30.6% Health Care, and 40.3% Social Sciences and Law. The study was carried out in accordance with the Declaration of Helsinki, and the protocol was authorized by the Bioethics Committee of University of Santiago de Compostela.

### 2.2. Measurements

#### 2.2.1. Compulsive Buying

Participants completed the German Addictive Buying Scale (GABS) [60] in its Spanish translated version [66] to evaluate their tendency to CB. The GABS includes 16 statements (e.g., “When I have money I have to spend it”, “For me, shopping is a way of facing the stress of my daily life and relaxing”, “I often feel a sudden, inexplicable urge to go out immediately and buy things that I want”) and ratings are made on a 4-point scale from 1 (strongly disagree) to 4 (strongly agree). The total score (ranging from 16–64) was used as an index of CB tendency. GABS showed good psychometric properties in previous research with samples of young Spanish participants [21,67]. Cronbach’s alpha for this sample was 0.82.

#### 2.2.2. Big Five Personality Traits

The BF personality traits were assessed using the Spanish version of the Revised NEO-Personality Inventory (NEO-PI-R) [68]. This is a self-report instrument which includes 240 items each rated on a five-point Likert scale from 0 (strongly disagree) to 4 (strongly agree) assessing neuroticism (e.g., “l am easily frightened”), extraversion (e.g., “I really like most people”), openness to experience (e.g., “I have a very active imagination”), agreeableness (e.g., “Most people I know like me”) and conscientiousness (e.g., “I am known for my prudence and common sense”). Raw scores were standardized as T-scores (M = 50, SD = 10) using the combined-sex adult norm reported in the manual. The NEO-PI-R has been used in Spain in previous studies in this field of study [46,69] and has shown suitable psychometric properties. Cronbach’s alphas for the BF personality domains were neuroticism (0.90), extraversion (0.87), openness to experience (0.82), agreeableness (0.85), and conscientiousness (0.88). 

#### 2.2.3. Self-Esteem

To assess self-esteem, we used the Spanish version [70] of Rosenberg´s Self Esteem Scale (RSES) [71], a ten-item questionnaire using a 4-point Likert scale (from strongly disagree to strongly agree). This scale contains five positively (e.g., “On the whole, I am satisfied with myself”, “I feel I have many good qualities”) and five negatively (e.g., “I wish I could have more respect for myself”, “I certainly feel useless at times”) worded items. The sum of the ratings assigned to each of the 10 items, after reverse scoring the negatively worded items, indicated one’s self-esteem level (scale 0–30). Higher scores correspond to higher self-esteem. RSES has previously shown adequate psychometric properties in other research carried out with Spanish samples [61]. In the current study, Cronbach’s alpha for the scale was 0.87.

### 2.3. Statistical Analysis

Statistical analyses were carried out using IBM SPSS statistical package version 29. The three regression analyses corresponding to the procedure described by Baron and Kenny [72] for mediator effects detection were conducted, using the ordinary least squares method, and with the variables entering simultaneously. Subsequently, to obtain a global picture of the relationships between BF personality traits, self-esteem, and CB, only those effects that reached statistical significance on the basis of the regression results were brought into the model which was subjected to empirical testing using path analysis. It was calculated with SPSS AMOS (version 29) software by using maximum likelihood as the estimation procedure. The goodness-of-fit of the model was estimated using the chi squared test, the χ^2^/df ratio, the comparative fit index (CFI), the goodness-of-fit index (GFI), the adjusted goodness-of-fit index (AGFI), the normed fit index (NFI), and the root mean square error of approximation (RMSEA).

## 3. Results

The principal objective of this study was to assess if self-esteem mediated the influence of the BF personality traits on CB. In line with the approach of Baron and Kenny [72], three regression equations were estimated: (a) regressing self-esteem on BF personality traits, (b) regressing CB on BF personality traits and (c) regressing CB on BF personality traits and self-esteem. To satisfy the mediation hypothesis, self-esteem should emerge as the main predictor of CB in the last regression analysis and the effect of the BF personality traits on CB must decrease.

The results of the first regression analysis (Table 1) confirm that the first criterion of Baron and Kenny [72] was met. Therefore, the BF personality traits are significant predictors of self-esteem (19.3% of explained variance). 

As to the second regression equation, the traits of neuroticism, conscientiousness and openness to experience are confirmed to predict CB at statistically significant levels (20.4% of explained variance). Finally, when the BF personality traits and self-esteem are included together (31% of explained variance), support is found for the mediation hypothesis proposed. Specifically, the value of openness to experience markedly decreases to the point of not being statistically significant. As for neuroticism and conscientiousness, the explanatory contribution of CB is reduced; although, in this case, coefficients are statistically significant. Sobel tests [73] show significant coefficients regarding the indirect effects of neuroticism (Z = 4.47, *p* < 0.001), extraversion (Z = −3.31, *p* < 0.001), openness to experience (Z = −2.39, *p* < 0.05), agreeableness (Z = −3.21, *p* < 0.01), and conscientiousness (Z = −4.17, *p* < 0.001) on CB. In sum, it could be concluded that self-esteem filters the influence of the BF personality traits in this behavioural problem. Neuroticism and conscientiousness exert, as well as the mentioned indirect effect, a direct influence on CB. 

With the ultimate purpose of obtaining a global representation of the relations between the BF personality traits, self-esteem, and CB, and guided by the results obtained in previous regression analyses, we have put forward a model that has been subject to empirical test from path analysis. Table 2 includes the means, standard deviations, and correlations between variables.

The results obtained from the correlation between CB, the BF personality traits, and self-esteem show that CB revealed a positive correlation with neuroticism (r = 0.40, *p* < 0.001) and negative correlation with conscientiousness (r = −0.31, *p* < 0.001) and agreeableness (r = −0.13, *p* < 0.01). The relationship between CB and self-esteem was negative and significant (r = −0.47, *p* < 0.001). Moreover, our results confirmed significant associations between the BF personality traits and self-esteem: negative associations with neuroticism (r = −0.30, *p* < 0.001) and positive association with conscientiousness (r = 0.28, *p* < 0.001), extraversion (r = 22, *p* < 0.001), agreeableness (r = 0.21, *p* < 0.001) and openness (r = 0.14, *p* < 0.01).

**Figure 1:**
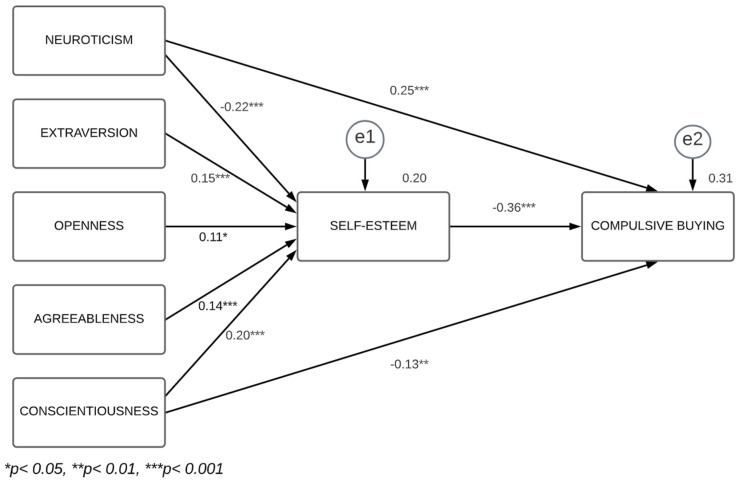
Final model with the relationships between the Big Five, self-esteem and compulsive buying.

The model to be tested envisages only the relations that reached statistical significance in the regression analysis results. Specifically, it is claimed that there is an indirect effect of the BF personality traits (neuroticism, extraversion, openness to experience, agreeableness and conscientiousness) on CB through self-esteem. The results of the path analysis, conducted using the maximum likelihood estimation as the procedure for parameter estimation, are presented in Figure 1. 

The goodness-of-fit indices show, in general, a good adjustment of the model to the data [χ^2^ (df) = 4.81 (3), *p* = 0.186; χ^2^/df = 1.60; CFI = 0.99; GFI = 0.99; AGFI = 0.97; NFI = 0.99; RMSEA = 0.03]. Thus, despite the fact that the statistic χ^2^ is significant, probably due to the high sample size (for more details, see Byrne [74]), the remaining fit indices are within commonly accepted ranges [75].

In sum, based on the analyses conducted, the mediating role of self-esteem is confirmed in relation to its influence on each and every BF personality trait for CB. Similarly, the domains of neuroticism and conscientiousness have, as well as the indirect effect mentioned above, a direct effect on the phenomenon under study.

## 4. Discussion

The study of which variables have a potential mediating role of the influence of the BF personality traits in CB has consolidated in the last decades as a suggestive and necessary research avenue. This study seeks to delve into the dynamic of influences between the BF personality traits and CB by adding self-esteem as the mediating variable. 

Generally, the results obtained confirm that self-esteem channels the effect of all and every one of the BF personality traits in CB. Specifically, the influence of extraversion, openness to experience, and agreeableness on CB are completely mediated by self-esteem, and the traits of neuroticism and conscientiousness present, as well as an indirect path, a direct effect on CB.

### 4.1. Explaining the Direct and Indirect Effects of Neuroticism in Compulsive Buying

The personal dimension of neuroticism emerges as that with the greatest influence—both directly and indirectly (through self-esteem)—on CB. The important direct and positive effect of neuroticism on CB found in this study is in line with not only the pioneering work of Mowen and Spears [38], but also with the findings of recent causal proposals by Otero-López and Villardefrancos [46], Aksoy et al. [10] and Tarka and Harnish [41]. There is, from a historical perspective, solid empirical evidence with regard to the link between negative emotions and CB [13,76,77]: this behavioral problem has been understood, as a primary response to negative events or emotions [64], as an attempt to repair previous negative emotional states [78,79]) or as craving for relief from negative internal states [80]. Be that as it may, on the basis of the result of this work, it may be argued that some of the facets of neuroticism (e.g., anxiety, depression, impulsiveness, vulnerability to stress) probably act, in some cases and under some circumstances, as triggers of CB, as the latter sometimes acts as a compensatory mechanism in dealing with negative feelings.

As for the path mediated by neuroticism (self-esteem acting as a channel for the influence of this trait on CB) the following pattern of influence emerges: high levels of neuroticism entail an important erosion of self-esteem which, in turn, increases vulnerability to CB. The precedent role of neuroticism and its influence on self-esteem (mediating variable) is consistent with the postulates in *The Five-Factor Theory of Personality* [26] which place traits at the base of the personality system (basic tendencies) and self-esteem occupies a second level. A person who scores high in neuroticism not only tends to experience more negative emotions but also sees the world and themselves through a lens of negativity, which leads to undermining self-esteem. In the previous literature, a close relationship between neuroticism and low self-esteem has been reported [49] and, also from a clinical perspective, psychological distress is linked to weak self-worth [81,82]. As for the influence of self-esteem on CB observed in the model, it should be mentioned that different empirical studies have reported that a negative self-worth contributes to predicting CB [15,36,65]. In this regard, there are a number of different hypotheses that can be found in the literature with regard to the function and/or usefulness, for some people, of CB: improving identity [83], a way to manage or enhance a poor sense of self [84] and bolstering one’s self-esteem [85].

The suitability of our model, with regard to neuroticism, becomes evident if we consider that there are a variety of works that place negative emotionality as the precedent, self-esteem as the mediating variable, and CB as the endogenous variable. Specifically, recent studies have found that self-esteem mediates the relation between stress and online CB [86], between trait anxiety and CB behavior [87] and between depression and CB [88]. 

Lastly, in an attempt to provide a theoretical foundation for the relevance of self-esteem in the neuroticism–CB link, it may be useful to consider the postulates of the self-enhancement theory [89]. This theoretical framework holds that people have a desire to protect and improve their feelings of self-worth and when this desire is frustrated, psychological distress emerges. Consequently, the hypothesis may be posited that the tendency of people vulnerable to CB to acquire material goods—particularly those linked to image—could be considered as an attempt to reach and maintain high self-esteem. Findings that compulsive buyers attach greater importance than those who do not present with CB to the life aspirations of image, financial success, and popularity [19,85] provides further support to this argument. 

In sum, this twofold route of influence of neuroticism on CB, found in this study, shows not only the tendency of compulsive buyers to purchase objects when they experience negative emotional states, but also that insecurity perceived at a personal level [90] may account for adopting a materialist view of life as a form of compensating for these feelings. 

### 4.2. Explaining the Direct and Indirect Effects of Conscientiousness in Compulsive Buying

As for the effect of conscientiousness on CB, it has been found that, although self-esteem channels part of its influence, it is also true that there is a direct effect on this behavioral problem. Thus, the direct route of conscientiousness with a negative effect on CB confirmed in this study is in line with previous research conducted on both college student samples [38] and the general population [46]. This type of result makes it possible to hypothesize that compulsive buyers, as well as having self-control deficits, may also find it difficult to organize and plan tasks. Furthermore, the relevance of this personality trait in the prediction of CB was made apparent in a very recent longitudinal study [91] which concluded that lower conscientiousness is associated with an increasing course of this behavioral problem.

Supplementary evidence of the importance of this personality dimension in CB can be derived from a number of studies which have discussed the inability to exercise self-control by compulsive buyers. Thus, for instance, Jiang and Shi [15] found that self-control has a predictive power not only for CB, but also for problematic Internet use and mobile phone use. Achtziger et al. [6] concluded that self-control moderates the debt–CB link and Nyrhinen et al. [92] show that low self-regulation in an online environment facilitates online shopping addiction.

As for the indirect route of conscientiousness on CB, our results confirm that the lower the conscientiousness, the greater the self-esteem deficit. Also, in line with other studies [3,24,36,60,61], the lower the self-esteem, the greater the vulnerability to CB. These findings are consistent with those in other studies, including that by Jiang and Shi [15] which suggests that decreased self-esteem and self-efficacy significantly predict CB, as well as other more recent studies [24,93] that found inverse relationships between CB and self-efficacy. Additionally, from the analysis of personal goals, the lack of confidence of compulsive buyers in their own capacity to attain intrinsic self-acceptance goals is confirmed [94], but it is also true that intrinsic self-acceptance and affiliation goals as opposed to extrinsic goals of image and popularity are protective factors against CB [19]. The low expectations of success and scarce progress in the attainment of personal projects in university students who are vulnerable to CB [67] are in line with the findings in this study. It could therefore be argued that those scoring low on a variety of conscientiousness facets (such as organization, perseverance, planning control, and perception of efficacy) also present a low self-worth which, in some cases, they seek to restore through CB. 

### 4.3. The mediating Role of Self-Esteem in the Link between Agreeableness, Extraversion and Openness to Experience and Compulsive Buying

The findings of this study also suggest that lower agreeableness, extraversion, and openness to experience were indirectly related to higher CB via self-esteem. In other words, low levels of agreeableness, extraversion, and openness to experience are associated with an erosion of self-esteem, which in turn is associated with an increased risk of CB. 

As far as agreeableness is concerned and in order to discuss the finding whereby this trait exerts an indirect influence through self-esteem, it should be noted that it is likely that some respondents who report low scores in agreeableness put their own interest ahead of the rest and frequently adopt an attitude of skepticism and distrust towards interpersonal exchanges. This attitude is ultimately detrimental to establishing solid social relations that are harmonious and conflict-free. Predictably, this non-agreeable behavior towards others means that the person perceives in other people unfriendly or suspicious attitudes, which will undoubtedly have a negative impact on their self-worth. Consequently, buying, particularly objects that enhance their personal attractiveness or their image in terms of the people they interact with, could be a way to try to restore that depreciated self-esteem. Supplementary empirical evidence is provided by the study by Roberts et al. [95] which confirms that the role of family conflict in driving CB operates through materialism and self-esteem. As for the mediated influence of self-esteem in the extraversion–CB link, it could be conjectured that, for some people, low scores in extraversion are accompanied by a reduced tendency to experience positive emotions, feelings of solitude, and a marked perception of low social support which, ultimately, increases their self-concept-related vulnerability. Buying may, once again, become a compensation mechanism. In this regard, previous research has confirmed that a feeling of loneliness is linked to CB [24,93] and that social support is a protection factor against CB [96]. Lastly, and as far as the indirect effect of openness to experience on CB through self-esteem is concerned, given the lack of alternative hypotheses in the literature, a tentative explication for the current finding could be that a lower comprehensiveness, depth, and permeability of conscience of the persons scoring low in this personality dimension could have an impact on a more unfavorable assessment.

In sum, the results of this study have shown the suitability of adding self-esteem as a mediating variable between the BF personality traits and CB. 

## 5. Conclusions and Practical Implications

In this study, so as to increase knowledge on the dynamics of influences between personality traits and dysfunctional buying behavior, a model of causal relations where self-esteem had a mediating role between the BF and CB underwent empirical verification. The results obtained allowed us to conclude that: (1) self-esteem is a necessary vehicle to channel the effect of each of the BF (neuroticism, extraversion, agreeableness, openness to experience, and conscientiousness) in dealing with CB-related problems and (2) that neuroticism and conscientiousness have (as well as an indirect effect through self-esteem) a direct effect on CB.

The practical implications as to what to do, based on the results, should consider self-esteem and the BF personality traits (especially, neuroticism and conscientiousness) as target goals that guide any preventive and intervention proposal. 

As for neuroticism and considering the important (direct and indirect) effect that this trait has in CB, several actions are recommended. Becoming aware of the relevant role that negative emotions have on CB, training in specific skills to identify them and acquiring resources to manage them efficiently are some healthy possibilities to minimize their impact on CB. Learning and applying techniques aimed at coping with states of emotional distress (e.g., relaxation, imagination, mindfulness, etc.) can also be an important step towards their management [8]. Specifically, recent evidence that a program focused on raising an individual’s level of emotional competence contributed to decreasing consumers’ inclinations towards CB [97] endorses our recommendation. Lastly, focusing attention on some facets of neuroticism such as anxiety, depression and vulnerability to stress that the previous literature has found to be strongly associated with CB [69] would contribute to reducing and/or mitigating their impact on this behavioral problem.

With regard to the influence that low conscientiousness has on both the undermining of self-worth and CB, it seems reasonable to think that by reinforcing some of its facets (deliberation, dutifulness, order, self-discipline, sense of competence), behavior control will increase and consequently this will be useful for the treatment and prevention of problems resulting from excessive buying. Thus, enhancing skills that contribute to resisting buying impulses (e.g., thinking before acting, planning buying, assessing before buying the need and usefulness of the item), identifying the emotional triggers that lead to CB, education on spending control (e.g., making shopping lists, avoiding the use of credit cards, setting spending limits) are just some recommendations.

As for self-esteem, as it is a core aspect of our model and channels the influence of each and every one of the BF personality traits on CB, it seems necessary to put forward action-oriented proposals. Some recommendations are (a) making the person aware of the many ways in which a low self-esteem (poor self-worth) may lead to CB (e.g., attention-seeking, instantaneous self-gratification, escaping negative feelings, improving image and the illusion of gaining identity, a need to socialize, seeking social approval and acceptance), (b) strengthening the feeling of personal worth/self-esteem by promoting interest in intrinsic goals (e.g., self-acceptance and affiliation) that would facilitate the basic needs of competence, relatedness and autonomy, and (c) designing intervention proposals based on the confirmed efficacy of some programs [98,99], the fundamental objective of which is the strengthening of self-esteem in people with buying problems. It is also necessary to take into account that any action aimed at enhancing self-esteem will not only reduce CB but will also weaken the “indirect” influence of the BF personality traits on this behavioral problem.

In sum, any action with assurances of efficacy should work on emotional instability, promote and educate on responsibility, and strengthen self-esteem. These notions should be echoed in the agendas of high-school and university health programs.

## 6. Strengths, Limitations and Future Perspectives

This study has some strengths that should be underlined: (a) it is the first study that has looked into the mediating role of self-esteem between the BF personality traits and CB, (b) the role of both the exogenous variables (BF personality traits) and the mediating variable (self-esteem) is consistent with hierarchical models of personality [26], (c) the order of influences that we have put forward (BF personality traits–self-esteem–CB) is in line with recent studies conducted on other behavioral additions [47,48]. It also has, however, some limitations that should be settled by future research. Firstly, the cross-sectional nature of the design does not allow the establishment of causal relations (this would require prospective and longitudinal studies aimed at shedding light on the dynamics of causal influences between the variables under study). As the self-report methods used in this study have a number of well-established biases (e.g., memory recall and social desirability), the use of other supplementary assessment procedures would also be desirable (e.g., buying diaries, third-party reports). The fact that respondents were university students limits generalization to other groups or developmental stages. In any event, and despite the limitations of the study, the findings of this study place us at a preventive and intervention stage where self-esteem must be seen as a potentially useful target on which to act to reduce the influence of the BF personality traits on CB.

It would be desirable that, given the important implications of our findings (tentative findings as this is the first study that looks at this causal proposal), future research should replicate this study in other types of samples, other age groups and other cultures to try to clarify the role of self-esteem with regard to its mediating role between the BF personality traits and CB and advance cumulative knowledge on the dynamic of influences between the BF personality traits and this behavioral problem. Adding and testing other mediating variables would undoubtedly be another interesting challenge for the future; thus, by way of example, the empirical conformation in different studies [100,101,102,103] that hardiness or meaning act as protective factors from other behavioral addictions (e.g., internet addiction and gambling disorder) means that these variables are potentially suitable to be added as mediating variables in new causal proposals. In short, any gain in understanding of the responsible variables and the dynamics underlying their influence will be welcome to channel the preventive and treatment efforts in dealing with a worrying and growing behavioral problem: CB.

## Figures and Tables

**Table 1:** Regression analyses performed to test for mediator effects of self-esteem.

	Big Five as Predictors of Self-Esteem *Beta*	Big Five as Predictors of Compulsive Buying *Beta*	Big Five and Self-Esteem as Predictors of Compulsive Buying *Beta*
Neuroticism	−0.22 ***	0.33 ***	0.25 ***
Extraversion	0.15 ***	0.02	0.07
Openness	0.11 *	−0.10 *	−0.06
Agreeableness	0.14 **	−0.07	−0.01
Conscientiousness	0.20 ***	−0.20 ***	−0.13 **
Self-esteem			−0.37 ***
*Explained variance (R^2^ adjusted)*	19.3	20.4	31

* *p* < 0.05; ** *p* < 0.01; *** *p* < 0.001.

**Table 2:** Correlations, means and standard deviations of all the variables studied.

	**1**	**2**	**3**	**4**	**5**	**6**	**7**
1. Compulsive buying	1						
2. Self-esteem	−0.47 ***	1					
3. Neuroticism	0.40 ***	−0.30 ***	1				
4. Extraversion	−0.04	0.22 ***	−0.06	1			
5. Openness	−0.08	0.14 **	0.03	0.28 ***	1		
6. Agreableness	−0.13 **	0.21 ***	−0.12 *	0.10 *	0.06	1	
7. Conscientiousness	−0.31 ***	0.28 ***	−0.31 ***	0.02	−0.04	0.11 *	1
*Mean*	29.81	20.69	59.90	48.04	50.90	38.07	41.53
*SD*	7.08	4.25	9.00	10.25	9.63	7.54	9.18

* *p* < 0.05; ** *p*< 0.01; *** *p*< 0.001.

## Data Availability

The data that support the findings of this study are available on request from the corresponding author. The data are not publicly available due to privacy restrictions.
